# Exploring the Characteristics and Outcomes of Centenarian Patients Admitted to Intensive Care Units: A Systematised Narrative Review

**DOI:** 10.7759/cureus.107636

**Published:** 2026-04-24

**Authors:** Carl Reyneke, Nattaya Raykateeraroj, Jemin Suh, Jacob Zand, Dong-Kyu Lee, Laurence Weinberg

**Affiliations:** 1 Anaesthesiology, Austin Health, Melbourne, AUS; 2 Department of Anesthesiology, Faculty of Medicine, Siriraj Hospital, Mahidol University, Bangkok, THA; 3 Department of Anesthesiology and Pain Medicine, Dongguk University Ilsan Hospital, Goyang, PRK

**Keywords:** centenarians, critical and intensive care, elderly in critical care, intensive care unit, oldest old

## Abstract

Centenarians are among the most rapidly expanding age demographics in Australia and are being admitted to intensive care units (ICUs) at historically high rates. Nonetheless, the current literature has not investigated the outcomes and pre-admission characteristics of this vulnerable population in sufficient depth to meaningfully guide clinical practice. This narrative literature review aimed to synthesise the existing literature on ICU admissions in centenarians and identify previously reported pre-admission characteristics and outcomes. This systematised narrative review employed a structured search strategy guided by the Preferred Reporting Items for Systematic Review and Meta-Analysis (PRISMA) principles and used the Population, Concept, Context (PCC) framework to aid in defining the scope. A systematic search strategy was performed in Medline (Ovid interface), with inclusion criteria aimed at capturing primary research that investigated centenarians admitted to critical care and was published in English. Covidence was used to screen articles via title, abstract, and full-text review. Key data were extracted from studies into a predetermined table. Analysis of the current literature suggested that although clinicians often perceive ICU admission of centenarians as a high-risk practice, certain patients may recover to their baseline functional levels. However, the existing literature is scant and limited by small sample sizes. Substantial knowledge gaps exist, particularly regarding the predictors of successful ICU outcomes, functional recovery, and quality of life outcomes. This narrative review demonstrates the need for large-scale, multicentre observational studies to influence clinical decision-making regarding centenarians in the ICU and to guide critical care triage policies for centenarians.

## Introduction and background

In recent decades, the life expectancy of Australians has steadily increased by 6.1 years for men and 4.2 years for women in the past 30 years. As of 2023, the life expectancy for males born in Australia was 81.1 years, whereas that for females was 85.1 years [1]. Moreover, the population of centenarians, individuals aged 100 years or older, has grown rapidly. Between 1998 and 2018, the number of centenarians has more than tripled, increasing by 215%, whereas the general population increased by 34% over the same timeframe [2]. In 2018, 4,198 centenarians were residing in Australia, and demographic projections suggest that this number is likely to continue to markedly increase. Similar trends have been observed globally. The Population Division of the United Nations estimated that the world centenarian population was 451,000 in 2015 and is projected to rise by 50-fold to more than 25 million by 2100, thereby highlighting a global pattern of population ageing [3]. This demographic shift, along with advancements in surgical and medical healthcare, has resulted in increased intensive care unit (ICU) admission rates among centenarians. In some surgical specialties, the admission rates for patients older than 90 years have surged by more than 50% [4]. Furthermore, data from international studies on patients with hip fractures suggest that centenarians experience disproportionately higher rates of ICU admission than nonagenarians and octogenarians [5]. Consequently, the expected rise in the number of centenarians requiring and receiving intensive care services presents both clinical and ethical challenges for healthcare systems, both globally and in Australia.

Centenarians represent a unique and understudied population within critical care. With advancing age, centenarians experience substantial physiological changes that influence their management within intensive care settings. Age-related alterations in the cardiovascular system, including concentric left ventricular remodelling and diastolic dysfunction, predispose this population to heart failure with preserved ejection fraction [6]. In a cohort of 188 Australian centenarian outpatients, high rates of hypertension (40.1%), heart disease (30.5%), cerebrovascular disease (23.5%), respiratory conditions (23.5%), ocular disease (70.6%), arthritis (58.1%), and osteoporosis (28.1%) have been reported [7]. The combination of visual impairment and sarcopenia increases the risk of falls and fractures in this population. Furthermore, centenarians experience immune-related changes associated with advanced age, a phenomenon known as immunosenescence. This increases the risk of infections, autoimmune disorders, and neurodegenerative diseases with advanced age [8]. One study showed that the majority of autopsied centenarian brains showed at least four neuropathologic findings, and the presence of multiple neuropathologies was strongly associated with dementia [9]. Estimates suggest that 25.4% of centenarians experience mild cognitive impairment and an additional 52.5% meet the clinical criteria for dementia [10]. These neurological changes increase the risk of ICU-related complications, including delirium and rapid cognitive decline. Consequently, the complex clinical needs of centenarians place a growing burden on intensive care services, particularly given their rapidly expanding population.

The decision to admit centenarian patients to ICUs presents both clinical and ethical complexities to specialists. Although chronological age is a poor predictor of prognosis, it frequently influences clinical decision-making regarding escalation of care [11]. Although many clinicians might believe that ICU admission is futile in this population, emerging research suggests that certain centenarian patients are able not only to survive ICU admission but also to return to their baseline function [12,13]. A continuing clinical conundrum is identifying the centenarian patients likely to benefit from intensive care. However, evaluating successful recovery in centenarians extends beyond patient survival alone. Quality of life outcomes, such as patient-reported outcome measures (PROMs) and patient-reported experience measures (PREMs), as well as functional recovery, often outweigh survival alone as measures of successful outcomes in older patients [14]. These findings highlight the need for a more nuanced, patient-centred approach to ICU triage and interventions in centenarians, reinforced by accurate prognostic tools and a robust base of evidence.

Although the centenarian population in Australia is steadily increasing, limited research has described the characteristics and outcomes of ICU admission in this vulnerable group. Whereas previous studies have examined the demographic expansion of centenarians, their rising use of ICU services, and their distinct health profiles, few studies have specifically explored the pre-admission characteristics and post-admission outcomes associated with ICU care in this cohort [2,5,7]. Information on key clinical indicators, such as mortality rates, ICU length of stay, post-discharge quality of life, and longer-term outcomes, remains scarce or has been retrieved from studies with very small sample sizes [11]. Research in larger centenarian cohorts is necessary to better understand the clinical challenges faced by this population and to improve the appropriateness and quality of their ICU care. Developing a greater understanding of centenarians’ characteristics and outcomes would support more informed decision-making, help optimise clinical practice, and guide the development of healthcare policies tailored to this unique and expanding population. Accordingly, this review aimed to synthesise current evidence regarding the outcomes and pre-admission characteristics of centenarians admitted to ICUs, thereby identifying key trends and gaps in the literature, and elucidating priorities for future research.

## Review

Methodology

Study Methods

This systematised narrative review followed a structured search strategy guided by the Preferred Reporting Items for Systematic Review and Meta-Analysis (PRISMA) principles to allow for enhanced transparency, without aiming to meet full systematic review requirements. The Population, Concept, Context (PCC) framework was followed to define the scope and structure of the review during the planning phase. A formal meta-analysis was not performed due to the heterogeneity of included studies, small sample sizes and variability in reported outcomes. Instead, the review aimed to provide a narrative synthesis of current evidence rather than a fully systematic or exhaustive analysis.

Types of Studies

Because of feasibility constraints, only primary research published in English was included, whereas protocols, planned studies, editorials, journal letters, dissertations, and articles written in any languages other than English were excluded. Although these criteria might have introduced language bias, this strategy was considered acceptable because this narrative was aimed at synthesising existing knowledge and identifying gaps in the literature, rather than influencing clinical practice.

Eligibility Criteria

Literature reporting data for patients 100 years or older at any time until 9 May 2025 was included. Data from primary empirical research were included, whereas abstracts, posters, editorials, journal letters, protocols for planned studies, and dissertations were excluded. The eligibility criteria included centenarians admitted to critical care settings, comprising ICUs, coronary care units, respiratory care units, burn units, and recovery units. Admissions to any non-ICU hospital wards or evaluations of centenarian outpatients were excluded. Abstracts unavailable in English were excluded from the review. If an abstract met the eligibility criteria, but the full text was not in English, the text was translated into English and included in the review. Limiting our search to only English-language sources might have introduced bias and limited the generalisability of the findings to non-English-speaking countries. However, this limitation was deemed acceptable, given that this narrative review is not intended to influence evidence-based practice. Inclusion and exclusion criteria are summarised in Table 1.

**Table 1:** Inclusion and exclusion criteria for the centenarian population, concept, context, and types of evidence. ICU = intensive care unit

	Inclusion	Exclusion
Population	Human participants over 100 years old (centenarians)	Human participants aged under 100 years old; animal studies
Concept	Studies that evaluate health-related outcomes, complications, and characteristics of centenarians admitted to the ICU; studies that collect any health-related data pertaining to the outcomes of centenarians admitted to the ICU	Studies that evaluate any non-health outcomes, complications, or characteristics of centenarians admitted to the ICU (e.g. economic characteristics)
Context	Exploring the characteristics and outcomes of centenarians admitted to the ICU; mortality rate; length of ICU stay; baseline health status; comorbidities; Post-discharge quality of life; ICU readmission rates; ICU or treatment-related complications	Exploring outcomes of centenarians admitted to non-ICU wards; exploring outcomes of centenarian outpatients
Types of evidence	Primary empirical research (e.g. randomised controlled trials, cohort studies, case reports, cross-sectional studies); full-text articles	Abstracts written in non-English languages; abstracts or posters; editorial articles and journal letters; protocols and planned studies; dissertations

Search Strategy

Medline (Ovid interface) was searched for eligible publications, and further literature was identified from the reference lists of eligible studies. The development of the literature search strategy included the use of Medical Subject Headings (MeSH) and keywords relevant to the centenarian population and ICUs (Table 2). Medline (Ovid) was selected as the primary database due to its comprehensive coverage of biomedical and critical care literature, making it well-suited to the clinical focus of this review. This literature search was originally conducted on May 9 2025, and was updated in January 2026 before submission to capture recent publications. While limiting our search to a single database may reduce the breadth of captured studies, this approach was considered appropriate for a systematised narrative review aimed at identifying key themes and gaps in the literature rather than achieving exhaustive study inclusion. This limitation was mitigated through reference list screening of included articles to identify additional relevant studies.

**Table 2:** Literature search strategy for Medline (Ovid interface) database.

#	Query	Results
1	Centenarians/	150
2	Frail Elderly/	17,384
3	centenarian*.ti,ab,kf.	2,710
4	supercentenarian*.ti,ab,kf.	146
5	super-centenarian*.ti,ab,kf.	9
6	semisupercentenarian*.ti,ab,kf.	10
7	semi-supercentenarian*.ti,ab,kf.	36
8	oldest old.ti,ab,kf	3,506
9	frail elder*.ti,ab,kf.	4,679
10	Critical Care/	63,713
11	intensive care units/ or burn units/ or coronary care units/ or recovery room/ or respiratory care units/	86,925
12	Critical Illness/	43,105
13	critical care.ti,ab,kf	50,636
14	intensive care.ti,ab,kf.	223,169
15	burn unit*.ti,ab,kf.	1,909
16	coronary care unit*.ti,ab,kf.	4,504
17	recovery room*.ti,ab,kf.	3,915
18	respiratory care unit*.ti,ab,kf.	211
19	critical* ill*.ti,ab,kf.	78,670
20	ICU.ti,ab,kf.	100,575
21	10 or 11 or 12 or 13 or 14 or 15 or 16 or 17 or 18 or 19 or 20	372,392
22	1 or 2 or 3 or 4 or 5 or 6 or 7 or 8 or 9	25,471
23	21 and 22	488

Screening Procedure

**Figure 1:**
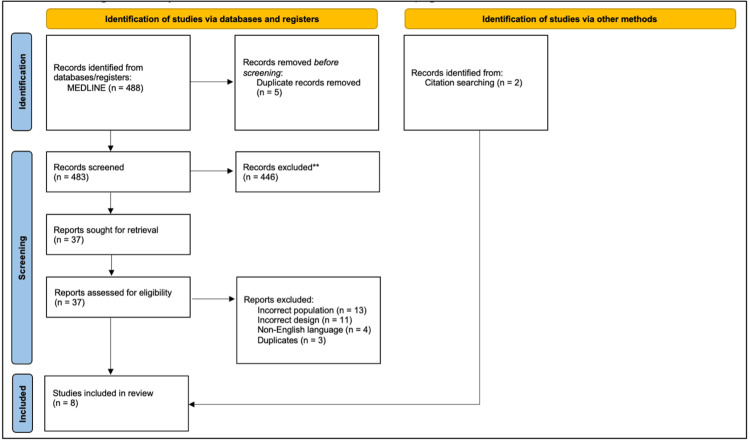
Preferred Reporting Items for Systematic Review and Meta-Analysis (PRISMA) flowchart illustrating the study selection process.

This review was performed in Covidence, an online systematic review platform. A three-step screening process involved title, abstract, and full-text review. Subsequently, full-text publications of all relevant and potentially relevant studies were retrieved and assessed for eligibility. Studies not meeting the inclusion criteria were excluded, and the reasons for exclusion were documented at the full-text stage. A PRISMA flowchart (Figure 1) was used to clearly illustrate the complete screening process.

Data Extraction

Eligible studies were entered into a custom-designed data extraction form to capture all relevant data from each study. Study selection and data extraction were performed by a single reviewer using the predefined eligibility criteria and a structured data extraction table (Table 3). Although formal inter-reviewer agreement was not assessed, uncertainties regarding study inclusion or data interpretation were resolved through discussion with co-authors to ensure consistency. The customised data extraction table was generated to efficiently highlight the evidence and achieve the aims of the review. The following data were extracted to meet the aims of the review: first author, year of publication, country of admission, study design, eligible population size, population demographics (e.g. sex and age), admission context, ICU mortality, ICU length of stay, post-discharge survival, ICU interventions, ICU complications, quality of life outcomes (including PROMs and PREMs), baseline functional status, comorbidities, and frailty scores.

**Table 3:** Data extraction table of searched literature meeting the inclusion and exclusion criteria. ICU = intensive care unit; MCA = middle cerebral artery

Author (publication year)	Ogawa et al. (2021) [5]	Wilson et al. (2000) [11]	Tosun et al. (2015) [12]	Bonfanti et al. (2018) [13]	Oliver et al. (2000) [15]	Cheung et al. (2016) [16]	Hogan et al. (2021) [17]	Bhandari et al. (2021) [18]
Country	Japan	USA	Turkey	Italy	England	Hong Kong	Ireland	USA
Study design	Retrospective	Prospective observational	Case report	Case report	Case report	Retrospective	Retrospective	Case report
Eligible population size	145	9	1	1	1	2	Not reported	1
Mean age (years)	Not reported	Not reported	111	101	113	Not reported	Not reported	105
Sex	Not reported	Not reported	Female	Female	Female	Not reported	Not reported	Female
Context of admission	Surgical (orthopaedic)	Mixed surgical and medical	Surgical (orthopaedic)	Medical (takotsubo cardiomyopathy)	Surgical (orthopaedic)	Surgical (orthopaedic)	Surgical (orthopaedic)	Medical (MCA ischaemic stroke)
ICU mortality (%)	Not reported	11.1	0	0	0	Not reported	Not reported	0
ICU length of stay (days)	Not reported	3.3 ± 1.1	1	Not reported	1	Not reported	Reported cumulative data	1
Post-discharge survival (months)	Not reported	Not reported	>6	>1	>9	Not reported	Not reported	Not reported
ICU interventions	Not reported	Reported	Not reported	Not reported	Reported	Not reported	Not reported	Reported
ICU complications	Not reported	Not reported	None	Not reported	Not reported	Not reported	Not reported	Not reported
Quality of life outcomes	Not reported	Not reported	Not reported	Not reported	Not reported	Not reported	Not reported	Not reported
Baseline functional status	Not reported	Not reported	Not reported	Not reported	Not reported	Not reported	Not reported	Not reported
Comorbidities	Not reported	Not reported	Reported	Reported	Reported	Not reported	Not reported	Reported
Frailty	Not reported	Not Reported	Not reported	Not reported	Not reported	Not reported	Not reported	Not reported

Risk of Bias

A structured risk of bias assessment was conducted for all included studies using the Risk of Bias in Non-randomized Studies of Interventions (ROBINS-I) tool for non-randomised studies (Table 4). Studies were evaluated across key domains, including confounding, selection bias, missing data, and outcome measurement. Each domain was classified as low, moderate, serious or critical risk of bias, with an overall judgement assigned based on the highest level of bias identified.

**Table 4:** Risk of bias assessment of included studies using the ROBINS-I tool. Risk of bias was assessed using the ROBINS-I tool. Domains were classified as low, moderate, serious, or critical risk of bias. Overall risk of bias was determined by the highest level of bias identified across domains. ROBINS-I = Risk of Bias in Non-randomized Studies of Interventions

Study	Confounding	Selection bias	Missing data	Outcome measurement	Overall risk of bias
Ogawa et al. (2021) [5]	Serious	Moderate	Moderate	Low	Serious
Wilson et al. (2000) [11]	Serious	Serious	Moderate	Low	Serious
Tosun et al. (2015) [12]	Critical	Critical	Serious	Moderate	Critical
Bonfanti et al. (2018) [13]	Critical	Critical	Serious	Moderate	Critical
Oliver et al. (2000) [15]	Critical	Critical	Serious	Moderate	Critical
Cheung et al. (2016) [16]	Serious	Serious	Serious	Moderate	Serious
Hogan et al. (2021) [17]	Serious	Serious	Serious	Moderate	Serious
Bhandari et al. (2021) [18]	Critical	Critical	Serious	Moderate	Critical

Results

Study Selection and Characteristics

A limited number of studies investigating centenarians admitted to ICUs were identified, consisting predominantly of case reports and small observational cohort studies. Only four observational studies included centenarian-specific data, with most studies focusing on highly selected populations. Sample sizes were small, with one study reporting outcomes in nine centenarians and others lacking a dedicated analysis of characteristics and outcomes of centenarians admitted to the ICU [5,11,16,17].

Admission Types

Most reported ICU admissions among centenarians occurred in the context of surgical management, particularly orthopaedic procedures following hip fractures [5,11,12,15-17]. Additional surgical admissions occurred following vascular bypass grafting, gastrointestinal surgery and post-trauma craniotomy [11]. Medical ICU admissions were less frequently described, including cases of gastrointestinal bleeding, takotsubo cardiomyopathy and acute ischaemic stroke [11,13,18].

ICU Length of Stay and Mortality

Reported ICU outcomes among centenarians were variable across studies. Case reports generally described favourable outcomes, including survival to discharge and return to baseline function [12,13,15,18]. One study reported a cumulative ICU length of stay of 13 days among 57 centenarians following hip fracture surgery [17]. However, the number of ICU admissions within this centenarian cohort was not specified, limiting interpretation.

In an observational study, ICU length of stay among centenarians was reported as 3.3 ± 1.1 days, compared to 2.5 ± 0.2 days in nonagenarians and 2.1 ± 0.1 days in octogenarians. ICU mortality was 11.1% in centenarians in this study, compared to 13.2% in nonagenarians and 7.3% in octogenarians. Post-discharge mortality was higher in centenarians (11.1%) than in nonagenarians (3.9%) and octogenarians (2.3%) [11].

Patient-Reported and Long-Term Outcomes

None of the identified studies reported PROMs or PREMs. Similarly, long-term follow-up data were limited, with only three case reports describing follow-up at one, six, or nine months [12,13,15].

Pre-admission Characteristics

Comorbidities were commonly reported in case studies, but none of the literature described standardised measures of pre-admission functional status or frailty. One study reported Simplified Acute Physiology Scores (SAPS), with no significant differences observed between centenarians (13.4 ± 2.1), nonagenarians (12.5 ± 0.3), and octogenarians (12.2 ± 0.1) admitted to the ICU [11].

ICU Interventions and Complications

Reporting of ICU interventions and complications was limited. While some case reports described supportive care measures, no studies systematically described complications such as delirium, nosocomial infections, or iatrogenic harm. One study reported Quantitative Therapeutic Intervention Scoring System scores, finding no significant differences between centenarians (40.0 ± 9.1), nonagenarians (31.7 ± 0.9), and octogenarians (30.7 ± 0.4) [11]. However, detailed descriptions of specific interventions were not provided.

Risk of Bias

All studies included in this review demonstrated either serious or critical overall risk of bias (Table 4). This was primarily attributable to small sample sizes and study designs limited to observational or case reports.

Discussion

Centenarians are an increasingly significant demographic in intensive care medicine. High prevalences of comorbidities and frailty complicate critical care management and contribute to prolonged hospital and ICU stays, as well as increased rates of complications such as cardiovascular and renal events in these patients. The presentation of centenarians to ICUs, whether planned or emergent, is likely to become more frequent with continued advancements in both life expectancy and medical technology. Nonetheless, insufficient studies have investigated ICU outcomes and pre-admission characteristics in this population. Both prospective and retrospective studies are scant, and most of the available literature has been limited to case reports. This review synthesised the current literature to identify key trends and deficiencies that should be investigated further. Given the heterogeneity of study designs, small sample sizes, and predominance of case-based evidence, a descriptive analysis of findings was undertaken rather than a quantitative pooling.

Admission Types

The predominance of orthopaedic surgery as a pathway to ICU admission likely reflects a convergence of age-related physiological vulnerability and clinical necessity. Centenarians are highly susceptible to fragility fractures due to osteoporosis and sarcopenia, alongside increased falls risk associated with visual and cognitive impairment [7]. Additionally, hip fractures often require surgical intervention, resulting in orthopaedic surgery being the predominant pathway to ICU admission in the centenarian population, given their limited physiological reserve and increased perioperative risk [5]. However, this trend is also influenced by selection bias, as surgically managed patients may represent a subset of centenarians with greater physiological reserve.

Length of Stay and Mortality Outcomes

Outcomes for centenarians after ICU admission are variable but are not consistently poor. Case reports identified in this population describe favourable post-discharge survival and return to baseline function. However, case reports in the context of centenarians are subject to publication bias, as patients with positive rather than negative outcomes are more likely to be documented. Observational data suggest that while ICU mortality in centenarians may not be significantly higher in younger elderly cohorts, post-discharge mortality appears to be elevated [11]. However, these findings are limited by small sample sizes and are susceptible to sampling bias. This underscores the need for studies in larger cohorts aimed at investigating the mortality rates of centenarians admitted to ICUs to produce more reliable data. Moreover, advances in ICU management in the time since this study may influence both ICU outcomes and clinicians’ thresholds of acceptable pre-admission physiological states for ICU admission, further complicating comparisons across studies [19].

Patient-Reported and Long-Term Outcomes

A striking deficiency in the literature was that no studies recorded any PROMs or PREMs. Both PROMs and PREMs are useful measures for improving the delivery and quality of patient-centred care in acute settings [20]. These measures are pertinent to centenarians, for whom functional recovery and maintenance of autonomy may be more important than objective measures of survival alone [21]. Additionally, the lack of long-term follow-up data outside of case reports limits the understanding of outcomes beyond hospital discharge, further restricting the clinical applicability of existing evidence.

Pre-admission Characteristics

Assessment of pre-admission characteristics indicated that case reports generally provided detailed information on the comorbidities of their centenarian patients. However, pre-admission functional status and frailty, widely recognised critical prognostic factors in geriatric intensive care, were not reported in any study [22]. The absence of these measures is a crucial deficiency in the literature. Future studies should incorporate validated, objective measures of frailty and functional status, such as the Clinical Frailty Scale (CFS) and Acute Physiology and Chronic Health Evaluation (APACHE) [22,23]. Severity of illness scores such as APACHE and SAPS should be carefully considered in elderly populations. Although the SAPS score considers age, it does not differentiate among patients older than 80 years. Additionally, SAPS does not consider frailty and functional status, factors widely considered to be more accurate outcome predictors than chronological age [24]. Future studies should consider these factors in investigating centenarians in the ICU to support contextualisation of reported outcomes and enable meaningful assessment of their clinical significance.

Risk of Bias

The overall quality of evidence identified in this review is low, with all included studies demonstrating either serious or critical overall risk of bias. This is predominantly attributable to a reliance on small observational cohorts and case reports, which are inherently prone to residual confounding and selection bias. Specifically, the absence of adjustment for key prognostic variables such as illness severity, frailty, and functional status limits the interpretability of reported outcomes. The large proportion of case reports in this review introduces a significant risk of publication bias due to a greater likelihood of favourable or unexpected outcomes being reported. As a result, the apparent survival and recovery observed in these studies may overestimate the true benefit of ICU admission in centenarians. Furthermore, selection bias related to ICU admission practices likely produces a highly selected cohort of comparatively resilient centenarians, thereby limiting the generalisability of findings to the broader population. These limitations restrict the ability to draw causal inferences from the data and reinforce the need for large-scale, multicentre observational studies with robust adjustment for confounding variables.

Gaps in the Literature

A major limitation of the literature as a whole is a lack of meaningful data from national databases and large observational cohorts. Only four observational studies included any data for centenarians admitted to ICUs. Moreover, only two of those studies reported ICU admission rates in centenarians after hip fracture surgery [5,16]. Combined analysis of those two studies suggested that ICU admission rates for centenarians after hip fracture surgery doubled, from 1.75% in 2016 to 3.54% in 2021. Although these studies came from comparable populations in Hong Kong and Japan, the poor accuracy in recognising trends when only two studies are available for analysis must be acknowledged. The lack of large-scale, multicentre, population-based studies limited our ability to reach generalisable conclusions to guide evidence-based practice. Additionally, the underreporting of ICU interventions and complications beyond mortality represents a significant gap in the literature. Obtaining information regarding the ICU interventions and complications that centenarians experience is critical for evaluating the potential benefits and harms of ICU admission and treatment, as well as providing patients and their families with realistic expectations for prognosis and recovery.

Future Directions

The shortcomings in the reviewed literature highlighted above indicate that comprehensive multicentre data are imperative to explore both the pre-admission characteristics and ICU outcomes in the centenarian population. Researchers should systematically document frailty, functional status, and comorbidities by incorporating standardised scoring systems such as CFS and APACHE on admission in future studies. This will allow for meaningful comparison across studies and improve prognostic assessment. Outcomes occurring both in the hospital (e.g., ICU length of stay, interventions, complications, and mortality) and after discharge (e.g., survival and function) should also be followed. Finally, researchers should consider subjective centenarian ICU experiences by using PROMs and PREMs reported by the patients themselves or their caregivers.

## Conclusions

Although centenarians represent a small cohort of patients admitted to intensive care, they are a growing population and are able to achieve meaningful benefits that outweigh the risks of ICU admission. However, the current literature evaluating these risks and benefits is substantially limited in scope and depth. This review identified a narrow scope of literature describing the pre-admission characteristics and outcomes experienced by centenarians admitted to ICUs. This included the context of presentation, admission rates and in-hospital mortality rates. Nonetheless, critical gaps exist in understanding the pre-admission health, functional outcomes, long-term outcomes and patient experiences, as well as the interrelationships among them in centenarians admitted to ICUs. Studies using high-quality, large-scale data are urgently required to ground the critical care of centenarians in evidence-based practice aligned with the values and goals of patients and their families. As ICU technology and populations evolve, the models governing how providers respond to and treat centenarians must evolve in turn to provide a personalised consideration of each patient beyond age alone.
